# Molecular and evolutionary insights into the structural organization of cation chloride cotransporters

**DOI:** 10.3389/fncel.2014.00470

**Published:** 2015-01-21

**Authors:** Anna-Maria Hartmann, Hans Gerd Nothwang

**Affiliations:** ^1^Systematics and Evolutionary Biology Group, Institute for Biology and Environmental Sciences, Carl von Ossietzky University OldenburgOldenburg, Germany; ^2^Neurogenetics Group, Center of Excellence Hearing4All, School of Medicine and Health Sciences, Carl von Ossietzky University OldenburgOldenburg, Germany; ^3^Research Center for Neurosensory Sciences, Carl von Ossietzky University OldenburgOldenburg, Germany

**Keywords:** CCCs, structure, phosphorylation, oligomerization, evolution, modeling, APC superfamily, synaptic inhibition

## Abstract

Cation chloride cotransporters (CCC) play an essential role for neuronal chloride homeostasis. K^+^-Cl^−^ cotransporter (KCC2), is the principal Cl^−^-extruder, whereas Na^+^-K^+^-Cl^−^ cotransporter (NKCC1), is the major Cl^−^-uptake mechanism in many neurons. As a consequence, the action of the inhibitory neurotransmitters gamma-aminobutyric acid (GABA) and glycine strongly depend on the activity of these two transporters. Knowledge of the mechanisms involved in ion transport and regulation is thus of great importance to better understand normal and disturbed brain function. Although no overall 3-dimensional crystal structures are yet available, recent molecular and phylogenetic studies and modeling have provided new and exciting insights into structure-function relationships of CCC. Here, we will summarize our current knowledge of the gross structural organization of the proteins, their functional domains, ion binding and translocation sites, and the established role of individual amino acids (aa). A major focus will be laid on the delineation of shared and distinct organizational principles between KCC2 and NKCC1. Exploiting the richness of recently generated genome data across the tree of life, we will also explore the molecular evolution of these features.

## Introduction

The solute carrier 12 (SLC12) gene family encodes electroneutral, secondary active cation-chloride cotransporters (CCCs). These proteins are located in the plasma membrane and mediate the symport of cations (Na^+^, K^+^) coupled with chloride (Cl^−^). The transporters play a crucial role in various physiological processes such as regulation of cell volume, directional ion transport across epithelial cells, secretion of potassium, and regulation of intracellular Cl^−^concentration in neurons (Payne et al., 2003; Adragna et al., 2004; Gamba, 2005; Kahle et al., 2008; Blaesse et al., 2009; Di Fulvio and Alvarez-Leefmans, 2009; Arroyo et al., 2013; Gagnon and Delpire, 2013; Kaila et al., 2014). The importance of CCCs is illustrated by their association with numerous human disorders including Anderman’s syndrome, Gitelman’s syndrome, Bartter’s syndrome (Simon et al., 1996; Kurtz et al., 1997; Vargas-Poussou et al., 1998; Kunchaparty et al., 1999; De Jong et al., 2002; Howard et al., 2002; Boettger et al., 2003; Gamba, 2005; Di Fulvio and Alvarez-Leefmans, 2009; Gagnon and Delpire, 2013), epilepsy (Rivera et al., 2002; Palma et al., 2006; Aronica et al., 2007; Huberfeld et al., 2007; Kahle et al., 2014; Puskarjov et al., 2015), neuropathic pain (Coull et al., 2003), spasticity (Boulenguez et al., 2010), brain trauma (Shulga et al., 2008), deafness (Boettger et al., 2002, 2003), autism (Lemonnier and Ben-Ari, 2010; Tyzio et al., 2014), and schizophrenia (Kim et al., 2012; Morita et al., 2014).

The SLC12 gene family belongs to the amino-acid-polyamine-organocation (APC) superfamily (Hediger et al., 2004, 2013; Höglund et al., 2011; Arroyo et al., 2013). Phylogenetic analyses suggest that gene duplication events—one likely occurring at the base of archeans and two at the base of eukaryotes—led to the diversification and neofunctionalization of paralogs CCC subfamilies (Hartmann et al., 2014). These subfamilies include the K^+^-Cl^−^ outward cotransporters (KCCs), Na^+^-K^+^-Cl^−^ inward cotransporters (NKCCs) and Na^+^-Cl^−^ inward cotransporters (NCCs), the polyamine transporter (CCC9), and the CCC interacting protein (CIP1; Gamba, 2005; Daigle et al., 2009; Di Fulvio and Alvarez-Leefmans, 2009; Arroyo et al., 2013; Gagnon and Delpire, 2013; Hartmann et al., 2014). The status of CCC9 as a true CCC member remains equivocal (Hartmann et al., 2014).

Additional whole genome duplication (2R hypothesis) and independent gene duplication events at the base of vertebrates led to their subfunctionalization into paralogs KCC (KCC1–4), NKCC (NKCC1+2) and NCC (all vertebrates), NCC2 (teleost fish) and NCC3 (amphibian and squamates) isoforms (Gagnon and Delpire, 2013; Hartmann et al., 2014). The presence of the additional teleost-specific NCC2 clade is probably due to an independent gene duplication event in the teleost fish lineage (Hiroi et al., 2008; Wang et al., 2009; Hartmann et al., 2014). Subsequent gene loss events resulted in the absence of KCC3 in birds, KCC4 in amphibians, and NCC3 in birds and mammals (Gagnon and Delpire, 2013; Hartmann et al., 2014). Similarly, multiple gene loss events at the base of vertebrates caused lack of CIP1 and CCC9 in extant vertebrates (Hartmann et al., 2014). In this review we will focus on the structure-function relationships of KCCs and NKCC/NCCs to delineate shared and distinct organizational principles. We will focus on KCC2 and NKCC1, as they are the main contributors to neuronal Cl^−^ homeostasis. Data from other family members will be included when they provide instructive information.

## Overview of the general CCC structure

Proteins with a common evolutionary origin share a protein identity of ~30% and often a similar structure and function (Murzin et al., 1995). This holds also true for the paralogs subfamily members KCCs and NKCCs that share a protein identity of ~25% and a similar structural organization (Gamba, 2005; Di Fulvio and Alvarez-Leefmans, 2009). As no X-ray structure of any full-length family member has been obtained so far, our current knowledge of their structural organization relies on biochemical and computational analyses. Hydrophobicity profiles according to the algorithm of Kyte-Doolittle (Kyte and Doolittle, 1982) suggest 12 transmembrane domains (TMs), flanked by intracellularly located N- and C-termini (Payne et al., 1996; Gerelsaikhan and Turner, 2000; Gamba, 2005). The number of predicted TMs (10–13), however, varies among different prediction algorithms (Gerelsaikhan and Turner, 2000; Di Fulvio and Alvarez-Leefmans, 2009). Experimental *in vivo* and *in vitro* analyses with deletion mutants of predicted TMs demonstrated that NKCC1 consists of 12 TM helices and intracellularly located termini (Gerelsaikhan and Turner, 2000; Gerelsaikhan et al., 2006). In addition, several extracellular loops (ECL) were confirmed by insertion of HA-tags in the ECL2 of KCC2, and ECL2, 3 and 4 of NKCC1 (Zhao et al., 2008; Acton et al., 2012; Somasekharan et al., 2012; Weber et al., 2014). A notable structural difference between KCCs and NKCC/NCCs concerns the position of a long extracellular loop (LEL) that is situated between TM5 and TM6 in KCCs, and between TM7 and TM8 in NKCCs/NCCs (Gamba, 2005). Several *N*-linked glycosylation sites have been identified in this LEL, which confirms its extracellular location (Hoover et al., 2003; Paredes et al., 2006; Weng et al., 2013). The intracellular location of various regulatory phospho-sites, targeted by cytoplasmic kinases and phosphatases, is also in full agreement with the current structural model (Rinehart et al., 2009; Monette and Forbush, 2012; Rosenbaek et al., 2014; Weber et al., 2014).

TMs are highly conserved among paralogs KCCs (74–80% identity) and NKCCs/NCCs isoforms (59–79% identity), likely due to their critical role in ion translocation (Payne et al., 1995, 1996; Isenring and Forbush, 2001; Gamba, 2005; Somasekharan et al., 2012). A similar degree of sequence conservation is observed for the C-terminus (KCC isoforms: 67–81%, NKCC/NCC isoforms: 48–56%). In contrast, the N-terminus is much more variable among paralogs KCCs (33–50% identity) and NKCC/NCC isoforms (13–28% identity) (Gamba, 2005). This terminus is, however, highly conserved among orthologous vertebrate family members (CCC sequences of the same isoform from fish to human), indicating a shared function of the N-terminus in orthologs. N-terminal splice variants of KCC1, 2, 3, as well as N- and C-terminal chimera of orthologous shark and human NKCC1 (74% protein identity) reveal that the termini are not involved in ion translocation and binding of loop diuretics (Isenring and Forbush, 1997, 2001; Tovar-Palacio et al., 2004; Mercado et al., 2005, 2006; Uvarov et al., 2007; Payne, 2009). Their main function therefore is regulation of transport activity by harboring sites for posttranslational modifications like phosphorylation (Rinehart et al., 2009; Monette et al., 2014; Weber et al., 2014). Furthermore, they contain structural motifs that are important for features such as isotonic activity of KCC2 (Bergeron et al., 2006; Mercado et al., 2006; Acton et al., 2012). The termini also partake in regulation of membrane expression (Lee et al., 2007, 2010; Zhao et al., 2008; Zaarour et al., 2012; Rosenbaek et al., 2014), basolateral and apical sorting in polarized cells (Carmosino et al., 2008) and oligomerization (Casula et al., 2001, 2009; Simard et al., 2004; Brunet et al., 2005; Parvin et al., 2007; Warmuth et al., 2009).

At present, the only available X-ray based structure is that of the C-terminus of the ancestral archean *Methanosarcina acetivorans* CCC (*ma*CCC; Warmuth et al., 2009). According to the alignment of Warmuth et al. (2009), the *ma*CCC C-terminus shares a protein identity of 12% to human KCC2 (*hs*KCC2) and 11.6% to human NKCC1 (*hs*NKCC1). This C-terminus consists of a mixed α/ß fold and two structurally related subdomains (Warmuth et al., 2009). Each subdomain is structured into a central five-stranded parallel ß sheet that is mostly connected via α-helices. The two five-stranded parallel ß sheets are orientated in an inverted repeat structure, whereby the ß5 and ß10 strands form counterparts. The precise organization of the other CCC regions is currently lacking. Yet, fruitful insights were recently obtained by modeling approaches building on the crystallized structures of AdiC (arginine/agmantine antiporter) and ApcT (broad-specific amino acid (aa) transporter), two related members of the APC superfamily (Somasekharan et al., 2012). Exploiting these data resulted in important structural insights concerning the organization of the TM and the translocation pathway as summarized in the following.

## Transmembrane domains: structural organization, ion translocation and binding of loop diuretics

Net transport of KCCs and NKCCs occurs usually at a stoichiometric ratio of 1K^+^:1Cl^−^ and 1Na^+^:1K^+^:2Cl^−^, respectively. The mechanism mediating coupled translocation of ions is not yet known. Concerning NKCCs, Hass and coworkers proposed an alternating access model with ordered binding and glide symmetry (Lytle et al., 1998; Payne, 2012). In this model, the extracellular ions bind in a determined order (Na^+^-Cl^−^-K^+^-Cl^−^) to an open-to-out (apo) state. The substrate bound state evolves to a fully occluded state to prevent diffusion of substrates. A conformational change causes transition to an inward-facing state, releasing the ions in the same order in which they were bound. The empty transporter returns back to the outward-facing state through a fully occluded state. Similar models were proposed for other crystallized members of the APC superfamily transporters such as AdiC and ApcT (Krishnamurthy et al., 2009; Kowalczyk et al., 2011). Structurally, these proteins display a twofold symmetry with TMs 1–5 and TMs 6–10 orientated in an inverted repeat structure (5+5 TMs). This organizational principle of two similar but inverted structural elements impart a pseudo-symmetry that provides a structural basis for the transport according to the alternative access model with outward- and inward-facing orientations (Kowalczyk et al., 2011). According to this model, TM11 and TM12 are located outside of this structure (Abramson and Wright, 2009; Fang et al., 2009; Shaffer et al., 2009; Kowalczyk et al., 2011). In an *hs*NKCC1 3D homology model, generated by the Forbush’s group using the structures of AdiC and ApcT as a template, the translocation pathway is lined up with TMs 1, 3, 6, 8, and 10, which are surrounded by the other helices. During transition from the outfacing structure to the occluded state, TM6 and TM10 appear to rotate and bend against TMs 1, 3, and 8 (Somasekharan et al., 2012). According to this 3D model, the translocation pocket forms at the level of Met382 (TM3) and TM6 (Payne, 2012). Intracellular release of substrates could be controlled by the intracellular loop (ICL) 1 that creates a flexible intracellular gate (Payne, 2012; Somasekharan et al., 2012).

**Figure 1:**
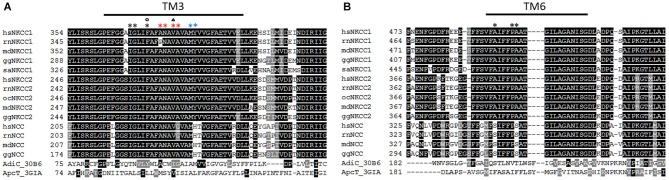
**Alignment of TM3 and TM6 of vertebrate NKCCs, NCCs AdiC, and ApcT. (A)** The alignment depicts residues involved in ion translocation in TM3. These residues are Met382, Tyr383 at the extracellular site (blue asterisk), Ala375, Asn376, Val378, and Ala379 building the translocation pore (red asterisk), and Phe372, Ile368, and Gly369 at the intracellular site (black asterisk). Residues that are important for Na^+^ affinity (circle) and K^+^ affinity (triangle) are also marked. **(B)** The alignment of TM6 highlights residues Pro496 and Ala497 whose mutation to Cys disrupts ion transport, and an Ala492Cys mutation that is sensitive to cysteine-modifying reagents. *hs* *(Homo sapiens)*, *rn* *(Rattus norvegicus)*, *md* *(Monodelphis domesticus)*, *gg* *(Gallus gallus)*, *sa* *(Squalus acanthias), oc (Oryctolagus cuniculus)*.

To verify the 3D homology model, Somasekharan et al. (2012) performed cysteine and tryptophan scanning mutagenesis of the highly conserved TM3 that lines up the translocation pathway (Figure 1). At the extracellular side of the translocation cavity, Met382 and Tyr383 are part of the extracellular gate, whereby the hydroxyl group of Tyr383 seems to take part in ion coordination. Interestingly, Tyr383 is highly conserved among vertebrate NKCC1 and NKCC2 whereas the K^+^ independent Na^+^-Cl^−^ cotransporter NCC exhibits a histidine instead of tyrosine (Figure 1). Within the translocation pathway, mutation of Ala379 to Cys, Leu, or Trp results in loss of function and mutation to Gly and Ser leads to decreased ion affinities. These data indicate an important role of these residues in ion translocation. Further residues like Ala375 and Asn376 are predicted to be exposed to the pore of *hs*NKCC1. At the intracellular side, Phe372 has a function in ion binding, and Ile368 and Gly369 are necessary for ion coordination at the cytoplasmic site. Interestingly, a tryptophan substitution of Phe372 affects the affinity for Na^+^, but not for K^+^, whereas a substitution of Val378 only affects K^+^ affinity. This observation supports the suggestion of the alternating access model with gradual binding of Na^+^-Cl^−^-K^+^-Cl^−^ (Lytle et al., 1998). The *hs*NKCC1 3D model also suggests that the bumetanide binding site is near the intracellular end of the pocket. Mutation of the intracellular gate residues Phe372 and Ile371 strongly decreased bumetanide binding and mutation of the extracellular gate residue Met382 completely blocked bumetanide binding (Somasekharan et al., 2012). These data are in line with previous observations that binding of bumetanide is dependent on the presence of all three ions and competes with the binding sites of one of the two Cl^−^ ions (Forbush and Palfrey, 1983; Haas and Forbush, 1986).

Further support for the 3D homology structure came from cysteine-scanning mutagenesis of the highly conserved TM6 that also lines up the translocation pathway (Dehaye et al., 2003; Payne, 2012). Both TM1 and TM6, which are counterparts in the two inverted repeat structures, consist of α-helices that are interrupted in the proximity of the binding site resulting in the α-helices TM1a+b and TM6a+b (Krishnamurthy et al., 2009). This binding site creates a polar environment for the coordination of the substrates due to main-chain hydrogen bonding (Yamashita et al., 2005). A cysteine mutation of the highly conserved Pro496 near the end of the interrupted helix or the Ala497 that is conserved among vertebrate NKCC1 and NKCC2 (equivalent to Pro487 and Ala488 in the experimentally analyzed rat NKCC1 (*rn*NKCC1); Figure 1) disrupts function indicating a role in the translocation pathway (Dehaye et al., 2003; Payne, 2012). A cysteine mutation in the middle of the TM6a helix (Ala492Cys; Ala488 in rat NKCC1) is sensitive to cysteine-modifying reagents in the presence but not in the absence of extracellular Cl^−^ (Dehaye et al., 2003). This could be due to different conformations of TM6 in the outward-facing and the occluded state of *hs*NKCC1 (Payne, 2012).

Based on the crystal structure of AdiC, closing of the extracellular gate might be due to a small rotation of TM6 and TM10 (Monette et al., 2014). To analyze the rotation and translocation, cysteine-mutations were inserted into TM10 (Pro676Cys, Ile677Cys, Asn680Cys) and into TM11 and TM12 (Ile730Cys, Ala734Cys, Ala735Cys, Trp733Cys). All three TMs are likely in close proximity. Disulfide cross-linking of TM10 and TM12 via Pro676Cys-Ala734Cys and Ile677Cys-Ala734Cys inhibits ion transport consistent with a movement of TM10 during occlusion (Monette et al., 2014). In contrast, cross-linking of Pro676Cys and Ile730Cys increased transport activity of *hs*NKCC1, probably due to a “lock” in an activated state. Likely, this activated state is physiologically induced by N-terminal phosphorylation (Monette et al., 2014). In the inactive state, Asn680Cys-Trp733Cys, Asn680Cys-Ala734Cys, and Ile677Cys-Ala735Cys cross-link to each other supporting their close proximity. As Asn680 is one helix further in the membrane compared to Ile677, Monette et al. (2014) suggested an inward movement of less than 5 Å of TM12 relative to TM10 during activation.

Previous biochemical analyses mainly exploited differences in functional properties between orthologous species or splice variants of NKCCs and NCCs to identify residues that are important for ion transport and affinities for diuretics (Isenring and Forbush, 2001; Payne, 2009, 2012). Thiazide-type diurectics and loop diuretics like furosemide and bumetanide are important pharmacological inhibitors of CCCs and frequently used to characterize the contribution of CCCs to physiological processes. Chimera exploiting different affinities for ions and diuretics between shark (*sa*NKCC1) and *hs*NKCC1 suggested that TMs but not the termini are responsible for the differences in affinities (Isenring and Forbush, 1997, 2001; Isenring et al., 1998a,b,c). TM2 was shown to influence Na^+^ and Rb^+^ kinetics, TM4 Rb^+^ and Cl^−^ kinetics, and TM7 Na^+^, Rb^+^ and Cl^−^ kinetics (Isenring and Forbush, 1997, 2001; Isenring et al., 1998a,b,c). In-depth analyses of TM2 demonstrated that an evolutionary change from Ala-Leu → Ser-Val (*sa*NKCC1_AL_ → *hs*NKCC1_SV_) is responsible for altered Na^+^ affinity and a Gly-Thr → Met-Met change (*sa*NKCC1_GT_ → *hs*NKCC1_MM_) for altered Rb^+^ affinity (Isenring et al., 1998b,c; Isenring and Forbush, 2001; Payne, 2012). Similar analyses on NKCC2 splice variants (NKCC2A, B and F), which differ in 23 aa in the TM2 and ICL1, provided hints to aa residues involved in determining Na^+^, Rb^+^ and Cl^−^ kinetics (Gagnon et al., 2005; Giménez and Forbush, 2007). Mutagenic analyses by converting the rabbit NKCC2B variant into the NKCC2F variant suggest, that three residues in TM2 (*rb*NKCC2B_ATG_ → *rb*NKCC2F_SVT_) and three residues in the ICL1 (*rb*NKCC2B_TAY_ → *rb*NKCC2F_MCV_) are important to turn Na^+^ and Cl^−^ affinities of NKCC2B into those of NKCC2F (Giménez and Forbush, 2007). The involvement of ICL1 suggests that this region is also embedded in the membrane (Giménez and Forbush, 2007). Further analyses revealed that residue 216 (*sa*NKCC2A_I216V_ or *sa*NKCC2F_V216I_) influences Rb^+^ affinity, residue 220 (*sa*NKCC2A_I220L_ or saNKCC2F_L220I_) the Na^+^ affinity, and residues 240 and 249 (*rb*NKCC2B_T240M,_ *rb*NKCC2B_T249M_ corresponds to *sa*NKCC2A_I216_ and to *sa*NKCC2A_T225M_) the Cl^−^ affinity (Gagnon et al., 2005; Giménez and Forbush, 2007).

Recently, a significant increase in K^+^ uptake over Cl^−^ uptake was reported for NKCCs under hypertonic condition (Gagnon and Delpire, 2010). Thus, a large fraction of K^+^ can apparently move independently of Cl^−^. This so called K^+^/K^+^ exchange is more pronounced for NKCC1 than for NKCC2. A chimera of the paralogs NKCC1 and NKCC2 revealed that ICL1 and ECL2 are important for this hyperosmotic K^+^/K^+^ exchange of NKCC1 (Gagnon and Delpire, 2010). These data indicate that TMs 2, 4, 7, ICL1, and ECL2 affect ion affinities in orthologous species. According to the 3D homology model, these TMs and loops surround the translocation pathway that is formed by residues of TMs 1, 3, 6, 8, and 10 (Somasekharan et al., 2012). Therefore, the results observed in chimera and mutants likely reflect indirect structural effects on the neighboring residues forming the translocation pathway (Payne, 2012). Unfortunately, analogous modeling of the 3D structure of KCCs and validating biochemical experiments are not yet available. However, Payne (1997) suggests a role of TM2 in the binding of cations as this TM is the most divergent transmembrane segment compared to the remaining eleven TMs.

## The large extracellular loop

In addition to short loops connecting TMs, KCCs and NKCCs/NCCs contain an LEL of ~100 aa residues in KCC2 and ~70 aa residues in NKCC1, as judged from hydrophobicity plots. KCC2 (Williams et al., 1999) and NKCC1 (Lytle and Forbush, 1992) are both glycosylated proteins and several *N*-linked glycosylation sites have been identified in the LEL (Hoover et al., 2003; Paredes et al., 2006; Weng et al., 2013). Detailed mutational analyses of these sites in KCC2 and NKCC1 have yet to be performed, but various data suggest an important role. Bulk deglycosylation of brain slices resulted in decreased NKCC1 transport activity (Ye et al., 2012). Unfortunately, no subcellular localization experiments were performed to examine whether reduced intrinsic NKCC1 activity or surface expression caused decreased Cl^−^-transport. Data from the related NCC and NKCC2 suggest a role of glycosylation in surface expression. Mutation of two aspartate-based glycosylation sites in the LEL of *rn*NCC (Asn404, Asn424) or *rn*NKCC2 (Asn442, Asn452) to glutamine decreased surface targeting and thereby transporter-mediated Cl^−^-flux in *Xenopus* oocytes (Hoover et al., 2003; Paredes et al., 2006). Mutation of both aspartates resulted in a stronger phenotype than mutation of a single residue. When the same mutational analysis was performed in the flounder NCC, which contains three *N*-glycosylation sites in the LEL, elimination of all three glycosylation sites reduced transport activity by 50% (Moreno et al., 2006). This decrease was quantitatively different from the 90% observed for the *rn*NCC double mutant (Hoover et al., 2003), indicating a different organization or role of the LEL between orthologous proteins.

In KCCs, glycosylation has only been studied in greater detail for KCC4. Four glycosylation sites (Asn312, Asn331, Asn344, and Asn360) are present in the LEL of the mouse KCC4 (*mm*KCC4) and enzymatic characterization of mutants revealed that Asn312 and Asn360 are linked to high mannose-sugars, whereas the two central residues, Asn331 and Asn344, contain complex-form glycans. The latter appear critical for surface expression of KCC4, as an Asn331Gln/Asn340Gln double mutant was retained in the endoplasmic reticulum (Weng et al., 2013). Mutation of only one of the aspartates was insufficient to block trafficking (Weng et al., 2013).

Beside the aspartates, the role of cysteines within the LEL was investigated. These cysteines are likely substrates of intra- or intermolecular disulfide bonds. In KCCs, the LEL harbors four cysteines which are highly conserved during evolution from *Drosophila* and fish to mammals. Mutation of any of these cysteines rendered *rn*KCC2 fully inactive in HEK-293 cells without affecting expression and surface targeting as judged by immunocytochemistry (Hartmann et al., 2010). This loss of transport function presumably reflects formation of erroneous disulfide bridges. Similar results were obtained for compound cysteine mutants. The same mutations, however, did not abolish transport activity of *mm*KCC4, the closest paralog (Hartmann et al., 2010). The LEL displays a higher sequence divergence (44.8%) between KCC2 and KCC4 than between the entire transporters (28%). Different requirement of cysteines might thus reflect distinct structural organization of their LELs. Swapping the LEL between the two paralogs revealed that KCC4 requires its own LEL to be transport active, whereas the KCC2 backbone tolerates LEL^KCC4^, as this chimera exhibits unchanged transport activity (Hartmann et al., 2010). These data suggest a different overall structural organization of KCC2 and KCC4 and a functional cross talk of the LEL with other, so far unknown regions of the transporter.

Diverging data were obtained for the role of cysteines in the LEL of NKCC1 from different species. Mutational analysis of five highly conserved cysteines in the LEL of *sa*NKCC1 demonstrated their requirement for transport activity. Substitution of any of them by glycine abolished transport activity (Jacoby et al., 1999). In contrast, substitution of four cysteines by serine in the LEL of *hs*NKCC1 decreased transport activity by only 20% (Somasekharan et al., 2013). Both studies were performed in HEK-293 cells and the different results are difficult to reconcile. They might either reflect different tolerance to the chosen substitutions (glycine vs. serine) or a difference between single substitutions (*sa*NKCC1) and a quadruple mutant (*hs*NKCC1). Finally, species-specific differences are also possible. Clearly, further comparative studies are required to settle this issue. It might also be interesting to study the role of these cysteines in other family members to gain insight into the evolutionary conservation of their functional role in the different family branches. To sum up, the organization and role of the LEL shows an amazing diversity between orthologs and paralogs. This is evidenced by the different functional consequences of aspartate mutations in the ortholog rat and flounder NCC, and by the different effect of cysteine mutations in the ortholog human and flounder NKCC1, or in the paralog rat KCC2 and KCC4.

The LEL was also thought to be involved in binding of diuretics. This was based on studies on chimera between family members with different affinities for the respective diuretic (Hoover et al., 2003; Castañeda-Bueno et al., 2010; Hartmann et al., 2010). As pointed out above, 3D model guided mutational analyses strongly argue for a binding of diuretics to the translocation pocket (Somasekharan et al., 2012). Thus, the observed changes in binding of diuretics after manipulations in the LEL likely reflect an indirect effect via changes in overall structural organization.

## Constitutive transport activity and the ISO domain

**Figure 2:**
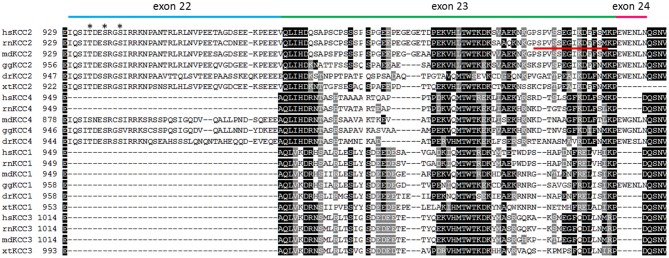
**Alignment of the C-terminus of vertebrate KCC isoforms**. The alignment depicts the amino acid residues encoded by exon 22 (blue bar), exon 23 (green bar), and exon 24 (pink bar) that are located in a part of the C-terminus (residues 929–1047 according to *rn*KCC2). Exons 22 and 24 are only present in all vertebrate KCC2 and placentalian KCC4 isoforms. The only exception is the presence of exon 24 in *gallus gallus* KCC1. Alignment of exon 23 indicates a higher variability among paralogs KCC isoforms. The phospho-acceptor sites Thr934, Ser937, and Ser940 that stimulate KCC2-mediated transport upon phosphorylation, are indicated as asterisk. The 15 amino acid long ISO domain located in exon 23 is indicated by a red line. *hs* *(Homo sapiens)*, *rn* *(Rattus norvegicus)*, *md* *(Monodelphis domesticus)*, *gg* *(Gallus gallus)*, *dr* *(Danio rerio)*, *xt* *(Xenopus tropicalis)*.

A defining feature of KCC2 is its high constitutive transport activity under isotonic conditions (Payne, 1997). This is essential to maintain a low (Cl^−^)_i_ in neurons. All other KCCs show low transport activity under isotonic conditions and require cell swelling for substantial transport activity. Several studies therefore attempted to identify the aa sequence in KCC2 that confers this unique functional property. In his original paper describing constitutive activity of KCC2 in HEK-293 cells, Payne alluded to a stretch of 41 aa residues in the C-terminus (KCC2b aa 929–970), as it was specific to KCC2 in sequence alignments of mammalian KCCs (Payne, 1997, 2009). An expanded alignment including recently obtained sequences from other vertebrate groups (Figure 2), however, reveals that this sequence is partially present in fish, birds and marsupial KCC4 as well. During evolution of placentalia, this sequence became part of an intron and thereby ceased to be translated at the ribosomes (Antrobus et al., 2012).

A detailed analysis using KCC2 and KCC4 mutants and chimera in *Xenopus* oocytes identified a 15 residue long sequence that conferred isotonic transport activity to KCC4 (Mercado et al., 2006). This 15 aa long sequence was called the ISO domain and is positioned between aa 1021–1035 in the *rn*KCC2b and thus proximal to the 41 aa residues, suggested by Payne (1997). The residues are encoded by exon 23, which is highly divergent in KCCs. A parallel study using the same approach obtained similar results, yet questioned whether this domain is required for isotonic activity (Bergeron et al., 2006). This conclusion was based on the fact that the absence of the ISO segment in KCC4 wild-type (wt) or KCC2/KCC4 chimera did not abolish transport activity under isotonic conditions.

A recent study in neurons provided compelling support for a role of aa 1022–1037 in constitutive transport activity. Transferring this sequence to KCC4 rendered this transporter constitutively active in hippocampal neurons, whereas its replacement by the corresponding KCC4 sequence abolished KCC2 constitutive activity. An important corollary of this study was the observation that the ISO-domain deprived KCC2 could be activated under hypotonic conditions, similar to KCC4. Thus, KCC2 has likely two functionally distinct domains, one KCC2-specific ISO domain, and a second domain, likely shared with other KCCs, conferring transport under hypotonic conditions (Acton et al., 2012). In the future, it will become important to study the evolution of the ISO domain, as its appearance likely has implications for the emergence of fast synaptic inhibition.

Mammalian NKCC1 and its closest paralog, NKCC2, exhibit both transport activity under isotonic conditions. Thus, isotonic activity is no specific trait of NKCC1 and has not been an issue in the field. However, a recent study addressed the difference in stimulation by hypertonic media between mammalian NKCC1 and NKCC2. Hypertonic conditions increased NKCC1 ~3-fold after heterologous expression in *Xenopus* oocytes, whereas NKCC2 was only stimulated ~2-fold (Gagnon and Delpire, 2010). Chimera and mutational analyses indicate that ICL1, TM2, and ECL2 of NKCC1 contribute to osmotic sensitivity (Gagnon and Delpire, 2010). Additional work will be required to characterize the precise interplay between these sequences and how they are affected by osmotic change.

## Oligomerization

Both KCC2 and NKCC1 form homo-oligomeric structures as evidenced by multiple biochemical and functional interaction studies (Moore-Hoon and Turner, 2000; Casula et al., 2001; Starremans et al., 2003; Simard et al., 2004; Blaesse et al., 2006; Parvin et al., 2007; Pedersen et al., 2007; Simard et al., 2007; Casula et al., 2009; Warmuth et al., 2009; Monette and Forbush, 2012). Monomers, dimers, and at least tetramers have been reported for KCCs, and monomers as well as dimers for Na^+^-dependent cotransporters (Starremans et al., 2003; Blaesse et al., 2006; Mahadevan et al., 2014). Additionally, both transporters have been suggested to oligomerize with other family members as well (Caron et al., 2000; Casula et al., 2001; Simard et al., 2007; Wenz et al., 2009). However until now, hetero-oligomerization has only been observed in heterologous expression systems. We will therefore limit our review to homo-oligomeric organization. The two major questions pertaining to oligomerization relate to its mechanisms and its requirement for transport activity. Currently, the answers to these questions appear more advanced for NKCC1.

Yeast two-hybrid studies revealed that the cytosolic C-termini of *hs*NKCC1 and *hs*NKCC2 contain self-interacting sequences (Simard et al., 2004). It was, however, unclear, whether they were important for intramolecular or intermolecular interactions. Elegant biochemical cross-linking studies with exchanges between the C-terminus of *rn*NKCC1 and the non-NKCC1 family members NKCC2, NCC, or CIP1 demonstrated the requirement of the C-terminal aa residues 751–998 for homo-dimerization of NKCC1 in HEK-293 cells (Parvin et al., 2007). In contrast, removal of the N-terminus did not affect dimerization (Parvin et al., 2007). Exploiting further this chimeric approach, the major interaction region was delineated to aa residues 806–814, with aa 842–912, aa 825–841, and aa 815–825 also supporting dimerization (Parvin and Turner, 2011). Substitution of any of four NKCC1-specific aa residues between 806–814 by the corresponding NKCC2 residues resulted in significantly impaired dimerization (Parvin and Turner, 2011). The involvement of the C-terminus in dimerization is supported by structural data of the archean CCC, which forms dimers in solution. The structure of the crystallized C-terminus suggests a relatively small hydrophilic dimer interface in which residues of α helices 1 and 2 in one subunit are in proximity to residues located in the loop connecting the β-strands 5 and 6 in the second subunit, and vice versa (Warmuth et al., 2009). An alanine substitution of Arg627 disrupts dimerization of *ma*CCC probably due to a loss of salt bridges that were formed by glutamate residues (e.g., Glu550 and Gl551) in α helix 2 of the adjacent C-terminus (Warmuth et al., 2009). Of note, mapping of the *rn*NKCC1 aa residues 806–814 to the archean CCC revealed that they are close to the putative dimer interface (Parvin and Turner, 2011). Thus, both experimental and structural data concur on C-terminus mediated dimerization of the Na^+^-dependent cotransporters. This conclusion is further supported by the observation of fluorescence resonance energy transfer (FRET), occurring between co-expressed NKCC1 clones tagged with CFP or YFP at the C-terminus (Pedersen et al., 2007; Monette and Forbush, 2012).

Concerning KCCs, first insights for oligomerization came from a pioneering study of the Alper’s group. They observed that an N-terminal truncated and transport inactive KCC1 variant, which was present at the surface, suppressed transport activity of co-expressed KCC family members in a dose-dependent manner in *Xenopus* oocytes (Casula et al., 2001). Subsequent work demonstrated that deletion of either the cytoplasmic N- or C-terminus did not impede oligomerization with full length KCC1, based on cross-linking studies in *Xenopus* oocytes and HEK-293 cells (Casula et al., 2009). These data point to the involvement of TMs for oligomerization. Unfortunately, a construct encompassing only the TMs and their connecting loops was not stringently tested. Its analysis would have been important to rule out the possibility that the observed oligomerization in the absence of either the N- or the C-terminus is merely reflecting the fact that either of the cytoplasmic regions is sufficient for oligomerization. Such a construct, however, is likely not well expressed at the surface. Another study using the yeast two-hybrid system observed dimerization of the full length C-terminus of KCC2 or KCC4 (Simard et al., 2007). In contrast, the C-termini of KCC1 and KCC3 failed to interact, similar to C-terminal fragments of the KCC2 or KCC4, truncated by ~100 aa residues from either of the two termini (Simard et al., 2007). For technical reasons, the cytoplasmic N-terminus and the TMs could not be analyzed in this system. Finally, a biochemical study using the rat hypothalamic cell line GT1–7 reported that removal of the final 28 aa of KCC2 resulted in a decrease of oligomers and an increase of monomers (Watanabe et al., 2009). Whether this decrease was due to lack of residues involved in dimerization or due to altered overall conformational changes of the truncated C-terminus was not investigated. Taken together, these data best fit a model, where oligomerization occurs via TMs and C-terminal sequences. Involvement of the C-terminus is in agreement with the aforementioned structural analysis of the C-terminus of the archean CCC (Warmuth et al., 2009). Contribution of the TMs is supported by the structure of AdiC (Fang et al., 2009; Gao et al., 2009). According to the crystal structure of this transporter at 3.2 Å resolution, TMs 11 and 12 provide the homodimeric interphase. Future studies should therefore address whether these two TMs fulfill the same function in KCCs. This would contrast the C-terminal mediated oligomerization in the Na^+^-dependent branch of CCCs. Yet, as pointed out by the Isenring’s group, interaction data are often difficult to interpret, as mutations and truncations may disrupt long-range interactions, thereby inducing erroneous conformations. In addition, biochemical analysis of oligomerization is challenged by the observation of rapid formation of KCC aggregates during purification, especially of overexpressed KCC2 in heterologous systems (Medina et al., 2014). These high molecular aggregates are for yet undefined reasons resistant to SDS (Medina et al., 2014). Both these features may lead to false negative and positive results.

With respect to the question whether homo-oligomerization is required for transport activity, three possibilities have to be distinguished: (i) Each subunit itself is a self-contained transporter and oligomerization is not required; (ii) Individual subunits mediate transport, but require oligomerization to adopt a transport active configuration; and (iii) Cooperation of both subunits is required, and substrate binding occurs to more than one subunit. The analysis of a concatemeric expression construct of NCC, consisting of a wt and a transport-impaired mutant, linked by a short spacer, revealed no change in transport activity, compared to a concatemer consisting of two wt NCCs (de Jong et al., 2003). Concatemers of transport impaired NKCC2 or AdiC transporter, another member of the APC transporter superfamily, with the respective wt transporter showed transport activities amounting to ~50% of a purely wt composed concatemer (Starremans et al., 2003; Fang et al., 2009). As transport activity was in neither case completely abolished, each individual NCC or NKCC2 polypeptide likely mediates substrate transport across the membrane, although an interaction of wt subunits from different tandem constructs cannot be excluded. FRET studies also demonstrated homomeric NKCC1 in the plasma membrane (Pedersen et al., 2007; Monette and Forbush, 2012). The previously reported functional interaction of different co-expressed splice variants of NKCC2 is therefore likely not reflecting cooperation of individual subunits for ion transport but rather due to altered trafficking or changes in functionally important interactions with the cytoskeleton (Plata et al., 1999). Whether dimerization of Na^+^-dependent transporters is required for transport activity and whether dimerization is regulated is presently unknown. FRET analyses and cross-linking analyses revealed a large movement of NKCC1 C-termini during activation processes. Blocking phosphatase 1, or exposure to low Cl^−^ hypotonic or zero K^+^ isotonic media stimulated NKCC1 transport activity, but decreased the intermolecular FRET signal (Monette and Forbush, 2012). This is likely due to a movement of TM12, which participates in dimerization, during activation of NKCC1 (Monette et al., 2014). The observed decrease in intermolecular FRET signals between the interacting C-termini during activation of NKCC1 indicates a loosening of the dimer during transport.

An important issue in the field is whether monomeric KCC2 is transport active or not. Several studies suggest that only oligomeric KCC2 is transport active. In the brainstem, an increase of higher molecular weight KCC2 complexes in SDS-PAGE paralleled activation of the transporter in auditory brainstem nuclei, suggesting a link between KCC2 oligomerization and transport activity (Blaesse et al., 2006). In contrast, another analysis using PFO-PAGE electrophoresis demonstrated oligomeric KCC2 already at P2 and revealed a tendency of KCC2 to form aggregates at high concentrations in heterologous expression systems (Uvarov et al., 2009). If aggregation holds true for native brain tissue as well, the developmental increase of high molecular weight forms of KCC2 in SDS-Page may partially be caused by the developmental increase in protein amount. Alternatively, the higher order KCC2 complexes observed in the PFO-PAGE might reflect association of the transporter with interacting proteins such as Neto2 or kainate receptors (see below). This type of hetero-oligomerization is likely lost in SDS-PAGE. Other studies reported a decrease of oligomeric KCC2 under conditions of decreased transport activity (Watanabe et al., 2009; Mahadevan et al., 2014). The dominant-negative effect of the N-terminal truncated, transport inactive KCC1, which does not affect surface expression of wt KCCs, supports the notion that oligomeric KCC2 is the only transport active form (Casula et al., 2001). Finally, Neto2, a KCC2 interaction partner, is required for KCC2-mediated Cl^−^ extrusion and associates preferentially with oligomeric KCC2 (Ivakine et al., 2013).

Due to the lack of crystal structure of an entire CCC protein, it is unclear why oligomerization should be required for transport activity. A clue might come from the observation that movement of TM12 is involved in inducing a transport active configuration in NKCC1 (Monette et al., 2014). According to the AdiC model, this TM can partake in dimerization as well. If this holds true, dimerization might help to appropriately position and move this TM in order to regulate transport activity. The assumption of oligomeric KCC2 as the sole transport active form does by no means preclude self-contained transporter subunits, as it was suggested for the Na^+^-dependent transporters and AdiC (Fang et al., 2009). Further studies including concatemers should be performed for KCC2 to settle this issue.

The correlation of oligomeric state and transport activity in combination with the variable ratio of monomeric to oligomeric form raised interest in mechanisms regulating oligomerization. The tyrosine kinase inhibitor genistein caused a significant shift towards monomeric KCC2 in GT1–7 cells, suggesting that tyrosine phosphorylation promotes KCC2 oligomerization (Watanabe et al., 2009). However, substitution of Tyr1087 by Asp, which mimics phosphorylated tyrosine, recapitulated the genistein effect. These results are therefore difficult to interpret and might indicate that the genistein-sensitive tyrosine kinase interacts rather with another kinase and not directly with KCC2. This notion is supported by the observation that both the tyrosine phosphorylation blocking agent genistein and the phospho-mimetic KCC2 mutant Tyr1087Asp caused a more positive reversal potential of GABA (Watanabe et al., 2009). Note, however, that the genistein and KCC2 Tyr1087Asp experiments were performed in different cell types (hippocampal neurons vs. GT1–7 cells), which might entail different effects of tyrosine phosphorylation. Finally, a recent analysis indicates that the tyrosine phosphorylation of KCC2 leads to an increased degradation of the transporter, both in HEK-293 cells and hippocampal neurons (Lee et al., 2010). Unfortunately, the role of tyrosine phosphorylation for oligomerization was not addressed in this study. The total amount of KCC2 parallels tyrosine phosphorylation of the transporter during brain development, as probed by the anti-phosphotyrosine antibody 4G10 (Stein et al., 2004). Tyrosine phosphorylation of KCC2 therefore does not correlate with the reported developmental increase of the oligomer to monomer ratio of KCC2 (Blaesse et al., 2006). Taken together, regulation of KCC2 oligomerization by tyrosine phosphorylation remains an open issue.

Localization to membrane microdomains may represent another factor involved in oligomerization. Two studies observed KCC2 in both membrane rafts and non-rafts (Hartmann et al., 2009; Watanabe et al., 2009). Analysis of the developing mouse brainstem revealed that monomeric KCC2, which prevails in the perinatal brainstem, mainly localized to membrane rafts. In the adult mice brainstem, KCC2 exists as monomers and oligomers and was recovered in both membrane rafts and non-rafts. The correlation between the developmentally increase of oligomeric KCC2 and the appearance of the transporter in non-rafts suggests that oligomeric KCC2 partitions to non-rafts. Yet, further biochemical and high-resolution microscopy are required to clarify the role of membrane rafts for KCC2 activity and organization. Finally, kainate receptors form high molecular weight hetero-oligomeric complexes with KCC2 (Mahadevan et al., 2014). Intriguingly, in mice lacking GluK1/2, an increase of monomeric KCC2 and a decrease in oligomeric KCC2 was observed (Mahadevan et al., 2014). It thus appears that kainate receptors influence the monomer to oligomer ratio of KCC2. It is currently unknown whether the interaction assists formation or promotes stability of KCC2 oligomers. It will be therefore interesting to study whether kainate receptors and KCC2 already interact in the trafficking pathway or only at the plasma membrane.

## Phosphorylation

**Figure 3:**
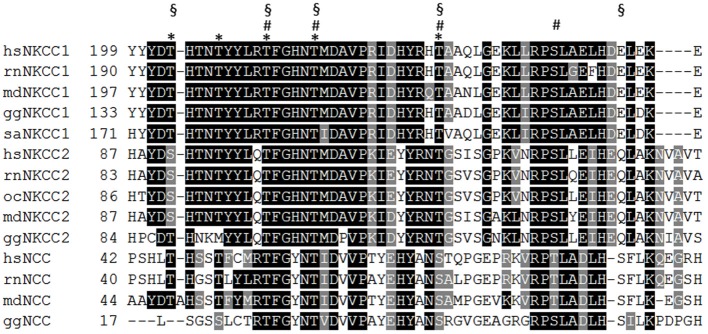
**Alignment of the N-terminus of vertebrate NKCC and NCC isoforms**. WNK-SPAK/OSR1 mediated phosphorylation of several threonine and serine residues that are located in a part of the N-terminus (residues 199–253 according to *hs*NKCC1). *sa*NKCC1: Thr175, Thr179, Thr184 Thr189, Thr202 are indicated as asterisk; *oc*NKCC2: Thr99, Thr104, Thr117, Ser126 are indicated as hashes; *hs*NCC: Thr46, Thr55, Thr60, Ser73, Ser91 are indicated as paragraphs. *hs* *(Homo sapiens)*, *rn* *(Rattus norvegicus)*, *md* *(Monodelphis domesticus)*, *gg* *(Gallus gallus)*, *sa* *(Squalus acanthias)*, oc *(Oryctolagus cuniculus)*.

Phosphorylation of the transporters has proven to be a powerful regulatory mechanism. Several excellent reviews have been recently published on this issue (Kahle et al., 2013; Alessi et al., 2014; Medina et al., 2014) and we will therefore focus on general principles, neglected issues, and open questions. Initial data proposed an elegant model in which KCC2 and NKCC1 activities were reciprocally regulated by phosphorylation. In *Xenopus* oocytes, coexpression of WNK3 (with no lysine kinase 3) with CCCs resulted in activation of NKCC1 and inactivation of KCC1 and KCC2 (Kahle et al., 2005). Subsequent analysis demonstrated that the action of WNK family members in combination with their functionally redundant downstream targets STE20/SPS1-related proline/alanine-rich kinase (SPAK) and oxidative stress responsive kinase (OSR1) results in phosphorylation of a cluster of threonines and serines in the N-terminus of NKCCs and NCCs (Darman and Forbush, 2002; Vitari et al., 2006; Richardson et al., 2008; Kahle et al., 2013; Alessi et al., 2014). The number of these phosphorylated aa residues varies between different family members, as they are not strictly evolutionary conserved among paralogs and orthologous family members (Figure 3). Phosphorylation of these residues is triggered by hypotonic low Cl^−^ conditions or a reduction in cell volume and results in stimulation of NKCC/NCC transport activity. For NKCC1, this activation was shown to be caused by conformational changes via a long-range interaction between the N-terminal phospho-site and other parts of the transporter (Darman and Forbush, 2002; Pedersen et al., 2007; Monette and Forbush, 2012). In addition to this intrinsic activation of NKCC1, a recent analysis of NCC revealed that phosphorylation of these residues increases surface expression by reducing internalization (Rosenbaek et al., 2014). Noteworthy, a serine/threonine phospho-dead NKCC1 mutant was reported to be expressed at the cell surface of HEK-293 cells (Somasekharan et al., 2013). Although no quantitative analysis of surface expression was performed, the result indicates phosphorylation-independent surface expression (Somasekharan et al., 2013). This observation raises the questions whether the consequences of N-terminal phosphorylation are different for paralogs NKCC/NCC isoforms and whether specific phosphorylation codes exist to trigger either intrinsic activation or surface expression.

In contrast to NKCC/NCC, regulation of KCCs by the WNK-SPAK/OSR1 signaling pathway involves the N-terminal Thr6 (numbering belongs to *rn*KCC2a) and Ser96 (KCC3A specific), and the C-terminal Thr906 and Thr1007 (numbering belongs to *rn*KCC2b) (Rinehart et al., 2009; Inoue et al., 2012; Kahle et al., 2013; Melo et al., 2013; de Los Heros et al., 2014). Thus, the opposite action of the WNK-SPAK/OSR1 signaling system on the two CCC branches is associated with a strikingly different positioning of their phospho-sites. A recent elegant analyses using a NKCC1 dimer with a C-terminal incorporated FRET probe and chemical cross-linking studies demonstrated that the N-terminal phosphorylation in NKCC1 results in a movement of TM10 and TM12 relative to each other and a movement of the C-terminus (Monette and Forbush, 2012; Monette et al., 2014). It is thus not excluded that phosphorylation of N-terminal or C-terminal phospho-sites affects similar regions in KCC2 and NKCC1.

This simple and appealing model of a reciprocal effect of phosphorylation of NKCCs/NCC and KCCs, however, has undergone substantial modifications. Recent data identified additional phospho-sites and revealed a complex role of phosphorylation for KCC2 function. Analysis of the evolutionary highly conserved Tyr903 and Tyr1087 revealed that dephosphorylation of both sites (mimicked by mutation to Phe) increased cell surface stability (Lee et al., 2010). Mutation of either of the two residues had no effect. This is in agreement with the unchanged transport activity of a Tyr1087Phe mutant in *Xenopus* oocytes (Strange et al., 2000). A phospho-mimetic KCC2 Tyr1087Asp mutant is transport inactive in *Xenopus* oocytes (Strange et al., 2000), GT1–7 cells (Watanabe et al., 2009), and neurons (Akerman and Cline, 2006; Pellegrino et al., 2011). As this mutant displays normal cell surface expression (Strange et al., 2000; Akerman and Cline, 2006), Tyr1087 likely influences the intrinsic activity. Clearly, further studies are required to fully understand the role of tyrosine phosphorylation for KCC2 function.

Importantly, phosphorylation can also activate KCC2. Phosphorylation of Ser940 increases surface expression, KCC2 transport function (Lee et al., 2007), and membrane clustering (Chamma et al., 2012). Lee et al. (2007) also identified another KCC2-specific serine residue, Ser728, whose mutation to alanine resulted in increased KCC2 transport activity. It will therefore be interesting to analyze this phospho-site in more detail. Finally, mutational analysis of Ser937 or the neighboring T934 showed that phospho-mimetic substitutions increased KCC2 transport activity as well (Weber et al., 2014). Surface expression analyses suggest that their phosphorylation results in intrinsic activation of KCC2, which would contrast and complement the activation mechanism by phosphorylation of Ser940. These recent data demonstrate that phosphorylation can both activate and inactivate KCC2 mediated transport. In this respect, it is interesting to note that phosphorylation of Tyr903, Thr906, Thr1007, Tyr1087, which are evolutionary highly conserved in KCCs, are all associated with inactivation. In contrast, the three phospho-sites Thr934, Ser937, and Ser940, which increase KCC2-mediated transport upon phosphorylation, are encoded by exon 22 (Figure 2). This exon is exclusively expressed in the vertebrate KCC2 and non-placentalian KCC4 isoform (Payne, 2009; Weber et al., 2014). Since phosphorylation of these aa residues results in increased KCC2 activity, they might provide an adaptive and fast reacting safety system to cope with challenging situations in the nervous system. The high transport activity associated with phosphorylation of these residues, however, comes with high metabolic costs and compromises effective responses to volume or intracellular pH changes (Kaila, 1994; Payne, 2009; Kaila et al., 2014). Switching between basal and high transport activity might therefore be advantageous, as it also economizes on the energetic costs of protein synthesis. Finally, the different conservation of phospho-sites across paralogs likely assisted subfunctionalization of new paralogs.

The absence of the three phospho-sites Thr934, Ser937, and Ser940 in the placentalian KCC4 also sounds a note of caution to the interpretation of the frequently used C-terminal chimera between KCC2 and KCC4. The observed differences might merely reflect presence or absence of these regulatory sites. It also has to be kept in mind that most data concerning KCC2 phosphorylation were obtained from *in vitro* systems such as cell culture or slices. Their precise *in vivo* roles have therefore still to be explored. This will likely require transgenic mice with mutated phospho-sites. Another important research field in the future will be the functional characterization of various native phospho-sites identified by mass spectrometry. According to publically available databases such as PhosphoSitePlus and Phosida, numerous phospho-sites scattered along the entire polypeptide have been observed for both NKCC1 and KCC2. Their analyses might help to identify whether NKCC1 contains C-terminal located regulatory phospho-sites similar to KCC2. We also still lack the staurosporine (and likely N-ethylmaleimide (NEM)) sensitive phospho-site. Both agents have been implicated in KCC2 activation, but their precise target has remained elusive (Lauf and Adragna, 2000; Lauf et al., 2001, 2006; Khirug et al., 2005).

An emerging issue is the cross talk of phospho-sites with other sites in CCC and their context dependent action. In *sa*NKCC1, a Thr202Glu mutant could be activated by preincubation in low Cl^−^ hypotonic medium, but not by preincubation in hypertonic medium, and became even inactivated upon transition from a low Cl^−^ hypotonic medium to hypertonic medium, contrary to wt NKCC1 (Darman et al., 2001). These data suggest that alternative activation modalities differentially affect a given phospho-site. Furthermore, this phospho-site is altering the phosphorylation of Thr184/Thr189 in *sa*NKCC1 (Darman et al., 2001). In KCC2, phosphorylation of Ser940 was affected by two KCC2 variants, Arg952His and Arg1049Cys, which were recently genetically linked to human idiopathic generalized epilepsy (Kahle et al., 2014). Finally, action of the broad band serine/threonine kinase inhibitor staurosporine and NEM was shown to be a function of the phosphorylation state of Thr934 or Ser937 (Weber et al., 2014). Whereas both agents activated wt KCC2 or alanine mutants of these two aa residues in HEK-293 cells, their effect reversed for aspartate mutants. Identification of the interplay between different phospho-sites will be important with respect to the concept of pharmacotherapeutical targeting of KCC2 phosphorylation. This approach requires comprehensive understanding of the interplay between different phospho-sites under varying conditions.

## Summary

Recent years have witnessed important progress in our understanding of the structural organization of the CCC family. The initially proposed topology of 12 TMs and two intracellularly located termini was confirmed and valuable information about molecular mechanisms of ion translocation and regulation of transport activity has been gathered. Noticeable, 3D modeling based on the structure of the related AdiC and ApcT, as pioneered by the Forbush’s group, provides a promising tool to bypass the lack of a crystal structure for any of the family members. The 3D homology model confirmed the previously proposed alternating access model in which ions bind in an ordered way to the apo state. A conformational change via the intermediate fully occluded state leads to an ordered release of the ions in the inward-facing state (Lytle et al., 1998; Kowalczyk et al., 2011; Somasekharan et al., 2012). According to the model, the translocation pathway is lined up with TMs 1, 3, 6, 8, and 10. Biochemical cross-linking and FRET analyses of the TMs 3, 6, 10 and 11/12 confirm this model. The successful application of this approach so far holds the promise that the translocation mechanism can be resolved through the interplay between structural modeling and biochemical approaches. However, we have to bear in mind that inferring functional properties from data obtained for a different family member is difficult, as many differences have already been reported between paralogs and orthologous CCC family members.

The cytoplasmic termini are mainly involved in regulation of transport activity via oligomerization, phosphorylation, sorting, and membrane expression. X-ray analyses of the archean *ma*CCC revealed that the C-terminus consists of a mixed α/ß fold and two structurally related subdomains (Warmuth et al., 2009). The C-terminus forms a small hydrophilic dimer interface in which residues of α helices 1 and 2 in one subunit are in proximity to residues located in the loop connecting the β-strands 5 and 6 in the second subunit, and vice versa. Oligomerization via the C-terminus was demonstrated for vertebrate NKCCs/NCC. Concerning KCCs, our current picture is less clear. Both the C-terminus and TMs have been implicated in oligomerization and settling this issue requires further studies. As these analyses have to account for the inherent difficulties in biochemical analysis of KCC2 in heterologous expression system, FRET based approaches hold great promises. Finally, phosphorylation has emerged as a key regulatory mechanism. Initially, a reciprocal regulation with KCCs being inactivated and NKCCs being activated via phosphorylation was observed. Yet, it has become evident, that at least for KCC2, phospho-regulation is more complex, including activation by phosphorylation and functional cross talk between different phospho-sites. Interestingly the three residues Thr934, Ser937, and Ser940, involved in phosphorylation-mediated activation, are situated in an exon that only exists in vertebrate KCC2 and non-placentalian KCC4. These residues might therefore provide an adaptive and fast reacting safety system to cope with challenging situations in the nervous system.

## Conflict of interest statement

The authors declare that the research was conducted in the absence of any commercial or financial relationships that could be construed as a potential conflict of interest.

## Abbreviations

CCC: Cation chloride cotransporter
KCC: K^+^-Cl^−^ cotransporter
NKCC: Na^+^-K^+^-Cl^−^ cotransporter
NCC: Na^+^, Cl^−^ cotransporter
GABA: gamma-aminobutyric acid
SLC: solute carrier family
TM: transmembrane domain
ECL: extracellular loop
ICL: intracellular loop
wt: wild type
aa: amino acids
LEL: large extracellular loop.
